# Increased burst coding in deep layers of the ventral anterior cingulate cortex during neuropathic pain

**DOI:** 10.1038/s41598-021-03652-7

**Published:** 2021-12-20

**Authors:** Fernando Kasanetz, Thomas Nevian

**Affiliations:** 1 Department of Physiology, University of Bern, Bühlplatz 5, 3012 Bern, Switzerland; 2 Grupo de Neurociencias de Sistemas, IFIBIO Houssay – CONICET, Universidad de Buenos Aires, Paraguay 2155 piso 7, (1121) Buenos Aires, Argentina

**Keywords:** Chronic pain, Neuroscience

## Abstract

Neuropathic pain induces changes in neuronal excitability and synaptic connectivity in deep layers of the anterior cingulate cortex (ACC) that play a central role in the sensory, emotional and affective consequences of the disease. However, how this impacts ACC in vivo activity is not completely understood. Using a mouse model, we found that neuropathic pain caused an increase in ACC in vivo activity, as measured by the indirect activity marker c-Fos and juxtacellular electrophysiological recordings. The enhanced firing rate of ACC neurons in lesioned animals was based on a change in the firing pattern towards bursting activity. Despite the proportion of ACC neurons recruited by noxious stimuli was unchanged during neuropathic pain, responses to noxious stimuli were characterized by increased bursting. Thus, this change in coding pattern may have important implications for the processing of nociceptive information in the ACC and could be of great interest to guide the search for new treatment strategies for chronic pain.

## Introduction

Neuropathic pain is a common pathological condition affecting approximately 5–10% of the general population^1,2^. Currently available therapeutic strategies have limited efficacy^3,4^, highlighting the need for a better understanding of the mechanisms underlying the disease^5^. It is now widely accepted that the long lasting sensory, affective and cognitive consequences of neuropathic pain are triggered by neuronal plasticity mechanisms occurring at all levels of the nociceptive system, including the higher order cortical regions involved in pain perception^6–10^.

The anterior cingulate cortex (ACC) is a central component of the brain response to pain and is believed to be of particular importance for the affective and cognitive assessment of pain^11,12^. Functional imaging studies of the human brain consistently showed ACC activation in response to acute nociceptive events and surgical ablation of the ACC in patients suffering from persistent intractable pain relieved pain averseness^13,14^. In rodents, manipulations that reduce or eliminate ACC activity abolished the affective manifestation of acute and chronic pain^15–18^ and prevented the positive reinforcement associated with pain relief elicited by analgesic drugs in animals with ongoing neuropathic pain^19,20^.

Evidences accumulated during the last decade indicate that plastic changes in ACC neurons are involved in the sensory, affective and emotional consequences of neuropathic pain^21–23^. The findings include long term synaptic potentiation of excitatory synapses onto layer 2/3 pyramidal neurons^21,22,24,25^ and increased somatic and dendritic excitability^26,27^ and synaptic disinhibition^26^ of layer 5 cells in ventral ACC (vACC). This data, collected in reduced preparations, converge in a scenario of enhanced excitability of ACC microcircuits (but see ^28^), which is believed to drive aversion associated with neuropathic pain.

Recently, this idea was supported by in vivo studies showing higher spontaneous and pain evoked firing in deep layers of the dorsal part of rostral ACC in neuropathic and inflammatory pain models^29,30^. However, since animals were awake and behaving, it is difficult to rule out that the enhanced ACC activity obeys to a higher afferent input from the sensitized nociceptive pathway^6,8^. In anesthetized mice, where the nociceptive drive is diminished, ACC neurons of layer 2/3 showed heightened activity only several weeks after neuropathic lesion, concomitant with the expression of pain-induced anxiodepressive-like symptoms^31^, despite the fact that synaptic changes typically occur at an earlier stage^21,22,26,27^. On the other hand, whether nerve injury-induced plastic modifications on layer 5 pyramidal neurons of vACC^26,27^ are associated with changes of its activity in vivo has not been tested yet.

Thus, the consequences of neuropathic pain on ACC activity in vivo is incomplete and appears to be more complex than suggested. In addition, little attention has been devoted so far to evaluate how neuropathic pain affects the patterns of activity of ACC neurons^31^. Robust changes in information transmission can be achieved with little variations in the overall firing rate but coupled to increased burst coding^32,33^.

Here, we used the chronic constriction injury (CCI) of the sciatic nerve in mice as a model to investigate how neuropathic pain affected the in vivo activity of deep layer pyramidal neurons of the vACC. We examined the level of activity of vACC neurons while mice were freely behaving in their home cage using the indirect activity marker c-Fos. Then, to avoid confounding effects of the sensitized peripheral and spinal nociceptive drive, we analyzed the firing rate and pattern of deep layers vACC neurons under ketamine anesthesia.

## Results

### c-Fos expression is increased in the vACC of mice with neuropathic pain

**Figure 1:**
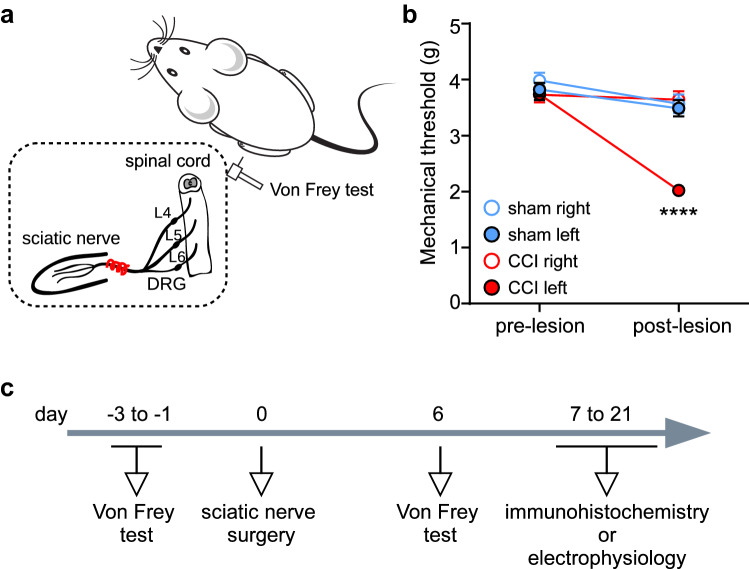
Chronic constriction injury (CCI) of the sciatic nerve induced mechanical allodynia of the injured hind paw. **(a)** CCI was induced by means of three loose ligatures on the sciatic nerve of the left hind paw. The lesion-induced allodynia was estimated from the mechanical threshold necessary to evoke paw withdrawal using an electronic Von Frey aesthesiometer. **(b)** After surgery, there was a reduction in the mechanical threshold of the injured (but not the contralateral) hind paw in CCI animals compared to sham (CCI *n* = 22; Sham *n* = 22; **** *p* < 0.0001, 2-way ANOVA). **(c)** Timeline of the experimental manipulations.

To evaluate how chronic pain affects the activity of vACC neurons in vivo we used the chronic constriction injury (CCI) of the sciatic nerve model to induce neuropathic pain in mice (Fig. 1a). We performed electronic Von Frey testing before and after peripheral nerve ligation to evaluate the development of mechanical allodynia in CCI mice (Fig. 1c). Compared to sham, CCI animals showed a significant lower mechanical threshold in the injured paw after but not before the lesion (Fig. 1b; pre lesion: sham = 3.82 ± 0.12 g, *n* = 22; CCI = 3.74 ± 0.11 g, *n* = 22; post lesion: sham = 3.49 ± 0.15 g, CCI = 2.03 ± 0.08 g; RM two-way anova: interaction: F(1,42) = 58.8, *p* < 0.0001, ES = 0.58, treatment: F(1,42) = 31.98, *p* < 0.0001, ES = 0.75, time: F(1,42) = 129.4, *p* < 0.0001, ES = 0.64; Bonferroni's multiple comparisons test for treatment: *p* < 0.0001). In contrast, the mechanical sensitivity of the uninjured paw did not differ between groups (pre lesion: sham = 3.98 ± 0.14 g, CCI = 3.73 ± 0.14 g; post lesion: sham = 3.57 ± 0.17 g, CCI = 3.64 ± 0.15 g; RM two-way anova: interaction: F(1,42) = 2.25, *p* = 0.14, treatment: F(1,42) = 0.24, *p* = 0.62, time: F(1,42) = 5.19, *p* = 0.02, ES = 0.11; Bonferroni's multiple comparisons test for treatment: not significant).

**Figure 2:**
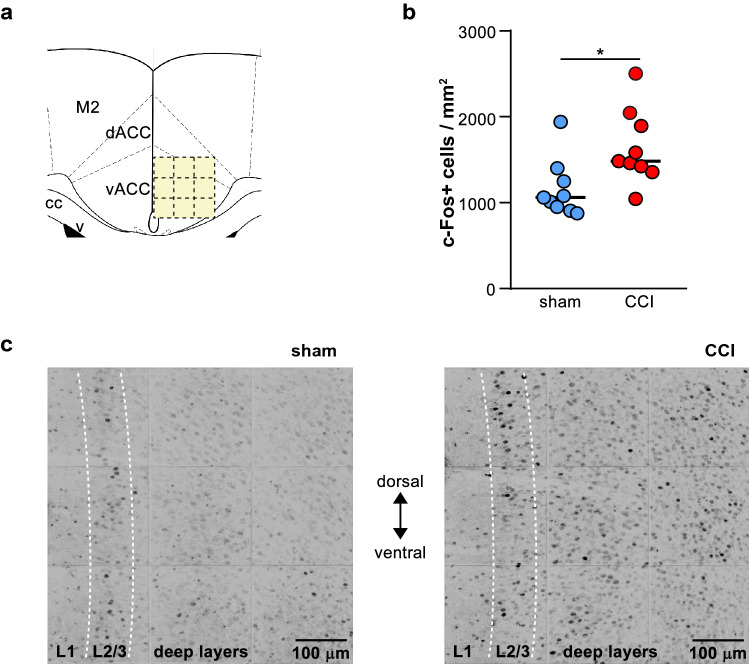
Enhanced number of c-Fos expressing cells in deep layers of the ventral ACC (vACC) in CCI-induced neuropathic pain. **(a)** Schematic diagram showing the grid of 9 squares (200 × 200 um each) covering the vACC used as region of interest for c-Fos expression analysis. M2, secondary motor cortex; dACC, dorsal ACC; cc: corpus callosum; v: ventricle. **(b)** The number of c-Fos immunoreactive neurons in deep layers of vACC was higher in CCI compared to sham mice (CCI *n* = 9; Sham *n* = 9; * *p* < 0.05, Mann Whitney test). **(c)** Representative confocal fluorescence microscopy images showing c-Fos immunoreactive neurons in sections of the CCA. Adjacent images were aligned to reconstruct the entire region of interest.

The proto-oncogene c-Fos is commonly used as an indirect marker of neuronal activity^34^. Previous studies have shown an increased number of c-Fos expressing cells in the ACC after common peroneal nerve ligation^35^ and sciatic nerve cuffing^36^ and also in the posterior cingulate cortex in CCI mice^37^. Here, we employed this strategy to map neuronal activation in deep layers of vACC induced by CCI surgery in freely moving animals (Fig. 2). Ten days after sciatic nerve lesion, significantly more c-Fos immunoreactive cells were observed in injured mice compared to mice that received sham surgery (Fig. 2; sham = 1062 (929—1324) cells/mm^2^, *n* = 9; CCI = 1483 (1389 – 1970) cells/mm^2^, *n* = 9; Mann Whitney test: U = 12, *p* = 0.011, ES = 0.85). This result supports the notion that the activity of vACC is affected in a state of neuropathic pain.

### Enhanced spontaneous firing rate of deep layers vACC neurons in CCI mice

The increased c-Fos expression in the vACC may reflect the augmented drive from a sensitized nociceptive pathway triggered by spontaneous pain or noxious events in the injured limb while the mice move in their home cage. Nevertheless, this may also indicate changes in the excitability of vACC microcircuits that alters the firing rate and/or firing pattern of deep layers vACC neurons.

**Figure 3:**
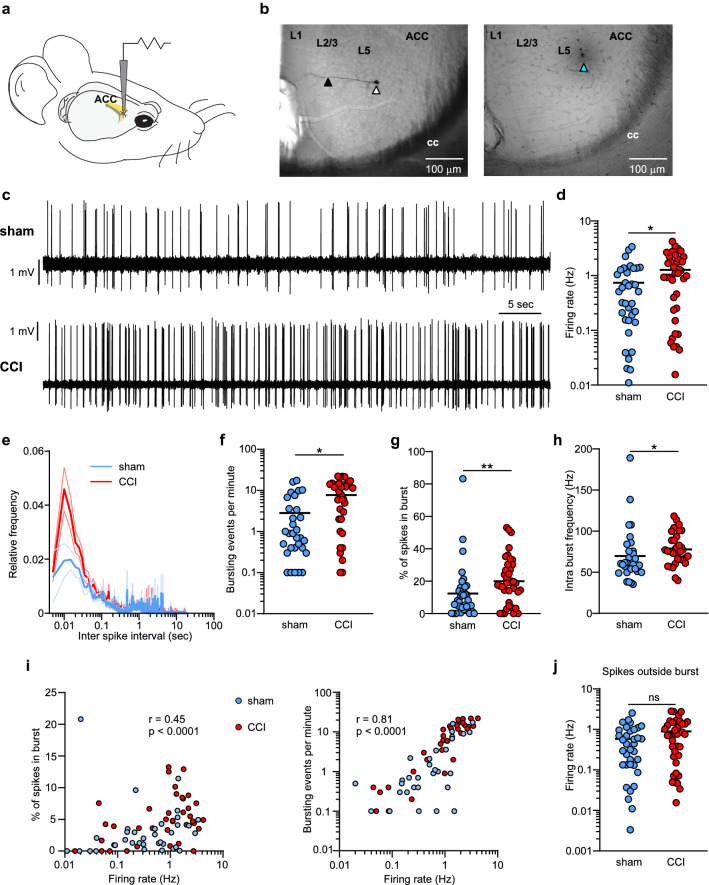
Increased spontaneous activity of deep layers vACC pyramidal neurons in mice with CCI of the sciatic nerve. **(a)** Schematic diagram showing in vivo electrophysiological recording of spontaneous activity in deep layers of vACC. **(b)** Representative microphotographs depicting recordings locations in vACC. Left: pyramidal neuron of layer 5 vACC labeled with biocytin during in vivo juxtacellular recording. Arrowheads indicate cell body (white) and apical dendrite (black). Right: pontamine sky blue spot deposited at the end of a recording session. The spot was used as a reference landmark to reconstruct recording locations of non-labelled cells. **(c)** Representative traces of one minute segments of spontaneous firing. Vertical deflections are single action potentials. **(d)** Enhanced spontaneous firing rate in neurons from CCI lesioned mice (CCI *n* = 38; Sham *n* = 35; * *p* < 0.05, Mann Whitney test). **(e)** Inter-spike histogram calculated from spontaneous activity. Thick and thin lines represent the mean and confidence interval (+ /- standard error), respectably. **(f–h)** Parameters related to bursting activity were increased in CCI mice (* *p* < 0.05, ** *p* < 0.01, Mann Whitney test). **(i)** For the overall population of neurons, bursting parameters were significantly correlated with spontaneous firing rate (Spearman correlation, *N* = 73). **(j)** Spontaneous firing rate outside burst was similar between neurons from sham and CCI lesioned mice (CCI *n* = 38; Sham *n* = 35; * *p* = 0.09, Mann Whitney test).

To gain insight into this, we investigated in more detail how CCI affects the firing activity of vACC by in vivo juxtacellular electrophysiological recordings in mice under ketamine-xylazine anesthesia (Fig. 3a-c), a condition of reduced nociceptive inputs^38^. Only a small proportion of cells recorded (3 out of 76) presented characteristics of fast-spiking interneurons (high frequency firing (> 5 Hz) and narrow spikes^39^) and were left out of subsequent analysis. Therefore, we assume that the vast majority of our recording belonged to pyramidal neurons, even though we cannot rule out the inclusion of a few cells from other interneuron types with pyramidal-like spikes. This way, we found that deep layers vACC neurons from nerve-injured animals had a significantly higher spontaneous firing rate than sham animals (Fig. 3d; sham = 0.51 (0.15—1.28) Hz, *n* = 35; CCI = 1.05 (0.21 – 2.07) Hz; *n* = 38; Mann Whitney test: U = 481, *p* = 0.04, ES = 0.64).

### vACC neurons from mice with sciatic nerve injury presented augmented spontaneous bursting activity

In addition to the overall firing rate, burst firing is an important feature of the neuronal code and can efficiently modulate the strength of synaptic transmission^32,33^. Several pathologies of the nervous system, including chronic pain, are associated with changes in the firing pattern of neurons with enhanced burst firing in various regions of the brain^31,40–42^. To investigate whether sciatic nerve injury modified the occurrence of bursting episodes in vACC neurons, we employed a maximum interval threshold method to define the occurrence of bursts. We chose this strategy because it has been shown to be efficient in detecting bursts in spike trains with relatively low overall firing rate containing sparse or little bursting, as it is the case for cortical neurons in vivo^31,43^. To do this, we first explored the inter-spike interval (ISI) histograms of spontaneous firing (Fig. 3e). The histograms of both control and CCI mice presented a smooth peak at around 1 Hz, corresponding to the slow oscillation between active and silent states induced by ketamine anesthesia. Interestingly, there was also a prominent peak in the high-frequency region with a half-decay at ~ 30 ms (Fig. 3e), which is consistent with the range of intra-burst ISI of pyramidal neurons. Based on this, we defined the criteria to identify bursts as episodes of at least 2 spikes with ISI < 30 ms, followed by a silent period longer than 100 ms. We quantified the properties of burst coding in vACC neurons and found that the rate of occurrence of bursting events (Fig. 3f; sham = 0.7 (0.1 – 3.4) events/min, CCI = 5.95 (0.37- 14.05) events/min; Mann Whitney test: U = 443.5, *p* = 0.013, ES = 0.67) and the proportion of spikes engaged in bursts (Fig. 3g; sham = 8.1 (2.5 – 16.0)%, CCI = 18.8 (6.2 – 30.2)%; Mann Whitney test: U = 421, *p* = 0.006, ES = 0.68) were higher in injured mice compared to controls. Notably, intra-burst firing rate was also increased in CCI mice (Fig. 3h; sham = 60.4 (51.1- 75.6) Hz, *n* = 31; CCI = 75.7 (65.2 -90.9) Hz, *n* = 33; Mann Whitney test: U = 327, *p* = 0.0127, ES = 0.68), indicating that also the quality of bursting transmission was affected by sciatic nerve lesion. Qualitatively similar results were obtained using slightly different criteria to detect bursts, like requiring at least 3 spikes in a train or removing the need for a pause after bursting (not shown).

Taken together, these results indicate that neuropathic pain induced increased spontaneous activity of vACC neurons distinguished by enhanced burst discharge. Thus, what would best describe the spontaneous activity of vACC neurons in CCI mice is the abundance of highly active burst-firing neurons. In agreement with this, there was a significant correlation between overall firing rates and bursting parameters (Fig. 3i; Spearman correlation, *N* = 73 pairs, firing rate vs % of spikes in burst: r = 0.45, *p* < 0.0001; firing rate vs bursting events per minute: r = 0.81, *p* < 0.0001). This notion was further illustrated when we removed the burst events from the analysis of the spontaneous firing frequency (Fig. 3j). In this case of only taking spikes outside the bursts into account, the average spontaneous firing rate was similar in sham and CCI (sham = 0.44 (0.13 – 0.95) Hz, *n* = 35; CCI = 0.76 (0.20 – 1.40) Hz; *n* = 38; Mann Whitney test: U = 512, *p* = 0.09).

Altogether, the results show that neuropathic pain was associated with an increase in the activity of deep layers vACC neurons even under conditions of reduced nociceptive drive. In particular, this is due to the appearance of a larger population of neurons highly active in burst mode in CCI mice.

### Increased burst coding of noxious stimuli in the vACC of CCI mice

**Figure 4:**
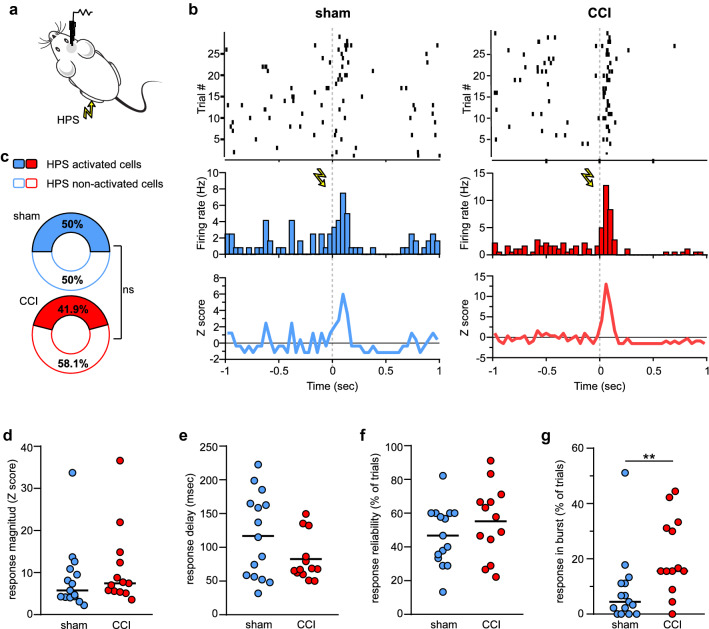
vACC neurons are activated by noxious stimuli. **(a)** Schematic diagram representing the electrophysiological recording of vACC while applying an electrical stimulus (HPS) to the operated hind paw. **(b)** Top: dot-raster representing firing activity before and after stimulus onset of two neurons activated by HPS. Each line correspond to a single trial aligned at the time of stimulus presentation (vertical dotted line). Middle and bottom: peri-stimulus firing histogram and Z score graph corresponding to the traces depicted above. **(c)** The proportion of HPS activated cells was similar between sham and CCI mice (ns: *p* > 0.05, Fisher exact test). **(d-f)** The magnitude, delay and reliability of the response to HPS in HPS-activated neurons were not modified by sciatic nerve lesion. **(g)** The proportion of trials with burst responses in HPS-activated neurons was higher in injured mice (** *p* < 0.01, Mann Whitney test).

Various studies in humans, non-human primates, rabbits and rodents have found that a portion of ACC neurons were activated by nociceptive stimuli^18,29,30,44–47^. We wondered how a condition of enhanced spontaneous bursting activity in neuropathic pain would influence the recruitment of vACC neurons by noxious stimuli. To test this, we recorded the firing activity of single units in response to electric stimulation of the hind paw (HPS) and observed that a fraction of cells reliable increased firing after stimulus onset (Fig. 4a-b). Setting a Z-score threshold of 2 we identified the neurons significantly activated by HPS (See methods, Fig. 4a-b) and found that half of vACC neurons from sham animals responded to the nociceptive stimulus. Surprisingly, the proportion of HPS activated cells did not change in injured mice (Fig. 4c; sham: 15 out of 30; CCI: 13 out of 32; Fisher’s exact test: *p* = 0.61). Furthermore, the response magnitude estimated from the Z score peak (Fig. 4d; sham = 5.74 (4.14 – 10.84), *n* = 15; CCI = 7.45 (5.47 – 13.62), *n* = 13; Mann Whitney test: U = 73, *p* = 0.27), response delay (Fig. 4e; sham = 116.8 ± 16.2 ms, CCI = 82.7 ± 9.4 ms; t-test: t(26) = 1.75, *p* = 0.25) and response reliability (Fig. 4f; sham = 46.7 ± 4.6%, CCI = 55.2 ± 5.9%; t-test: t(26) = 1.14, *p* = 0.26) were similar between sham and CCI mice. However, when looking at the pattern of activity during the response phase we found that neurons from neuropathic pain mice tended to fire in burst more often than cells from sham animals (Fig. 4g; sham = 4.4 (0 – 11.1)%, CCI = 15.5 (12.2 – 32.2)%; Mann Whitney test: U = 41, *p* = 0.008, ES = 0.79).

Hence, our data demonstrates that, at the single cell level, the most prominent feature of vACC responses to HPS in neuropathic pain is a shift towards encoding stimuli with bursts of spikes.

## Discussion

Neuropathic pain results from the expression of maladaptive plasticity within the nociceptive system. In particular, the remodeling of neural circuits in cortical areas involved in pain perception mediate affective/motivational consequences and mood alterations associated with chronic pain conditions^18,21–23,27,48–50^.

Neuropathic pain-induced synaptic and cellular adaptations have been extensively studied in the ACC^7^. We have recently shown that CCI of the sciatic nerve is associated with an increase in the excitability of pyramidal neurons in layer 5 of the vACC due to a loss of inhibitory inputs^26^ and a downregulation of dendritic hyperpolarization-activated-and-cyclic-nucleotide-regulated channels^23,27^. These changes are expected to result in increased activity of these neurons in vivo and make them more likely to respond to activation of the nociceptive system. Here we tested this hypothesis using different strategies to monitor neuronal activity in vivo. First, we used the expression of c-Fos as an indirect indicator of the level of neuronal activity while the animals moved freely in their home cages. As expected, we observed an increase in the number of c-Fos immuno-positive neurons in mice with neuropathic pain. This is consistent with previous studies using different models of neuropathic pain^35,36^. Considering that in our experiments the housing facilities, feeding procedures and handling of the animals were similar between the groups, higher c-Fos expression in CCI mice could be the result of changes in neuronal activity at rest, but also to events associated with a sensitization upstream to the ACC in the nociceptive pathway, such as spontaneous pain, allodynia in the injured paw triggered by locomotion or higher levels of anxiety^8,51^. Thus, it is not possible to discern if an enhanced intrinsic excitability of vACC microcircuits and/or increased nociception/anxiety-related afferent activity are responsible for the higher neuronal activation of deep layer ACC neurons. In order to gain insight on this, we performed electrophysiological recordings of spiking activity in mice anesthetized with ketamine / xylazine. Under these conditions, where nociceptive afferents loose influence on cortical activity^38^, neurons in the deep layers of the vACC showed a moderate increase in the spontaneous firing rate, suggesting that changes in the excitability of the vACC microcircuits could contribute to increased activity during neuropathic pain. Given that under ketamine anesthesia thalamo-cortical circuits present synchronous slow wave activity^52^, we cannot rule out that changes in the strength of the thalamic inputs have influenced the level of activity of ACC neurons in CCI mice. However, recent work showed that this does not appear to be the case, as the projections from the medial thalamus to the ACC were weakened in animals with neuropathic pain^28^. In conclusion, neuropathic pain is characterized by enhanced ACC in vivo activity which may result from the combination of local neuronal plasticity that renders ACC pyramidal neurons more excitable and a sensitized pain-related circuit that relays information to the ACC.

The most prominent feature in the activity of deep layers pyramidal neurons of the vACC in CCI mice was the increase in the occurrence of burst firing episodes. This happened in conjunction with a higher proportion of spikes involved in bursts and a larger firing frequency within bursts. This is consistent with findings from ex vivo recordings from layer 5 pyramidal neurons in the vACC^26^. The heightened burst activity would span the entire ACC microcircuit, as similar results were recently found in layer 2/3 neurons^31^. This widespread changes in activity pattern endow the ACC with a qualitatively different way of encoding information^33^. A burst of action potentials will more reliably cross a synapse, increasing the probability of triggering a postsynaptic spike, especially in synapses with a low probability of neurotransmitter release, such as many of the cortico-cortical and cortico-subcortical connections^33,53,54^. Thus, moderate changes in firing frequency associated with higher bursting activity could cause a substantial qualitative effect, altering the information transferred from the ACC.

Increased spontaneous bursting activity can have profound effects on information processing in the ACC. In fact, spontaneous activity, even under anesthesia, often exhibits a general similarity to activity evoked by sensory events^55–57^. Notably, here we found that higher spontaneous bursting activity was associated with more bursting events following HPS in mice with sciatic nerve injury. Bursting activity in response to sensory stimuli carries different information than single spikes^32^, suggesting that the encoding of nociceptive events by individual neurons in the ACC would be altered during neuropathic pain. Overall, the increased bursting activity could cause a deficit in the integration and filtering of information by the ACC networks. Consequently, this would limit the ability of the ACC to properly encode the affective value of nociceptive events (induced or spontaneous) in the context of previously acquired emotional memory and thus degrade the selection of appropriate behavioral strategies to cope with pain^12^.

Despite the overall increased activity of vACC neurons, that was mainly based on the enhanced bursting in CCI mice, this did not translate into an expansion of the pool neurons activated by HPS. Previous work found increases in the number of activated neurons or in the magnitude of their responses to nociceptive stimuli in dorso-rostral ACC^18,29^. This was observed using low intensity thermal or mechanical stimuli that in control animals produced mild responses, but triggered a paw withdrawal in animals with neuropathic pain. It is difficult to determine in these cases whether the higher cortical responses associated with allodynia are of local origin or driven by peripheral and spinal sensitization^6,8^. Here we used a high intensity stimulus to activate efficiently the nociceptive as well as the somatosensory system under anesthesia. We found that about half of the neurons were activated, a number that is in agreement with that reported in response to supra-threshold mechanical stimulation (inducing paw withdrawal) in awake animals^29^. Therefore, it is unlikely that in our work the anesthesia has masked a possible expansion of the HPS-activated neuronal assembly in injured animals. Perhaps the main effects of the increased excitability of ACC microcircuits are not related to changes in the ability of nociceptive stimuli to activate individual neurons, but rather to the ability to recruit the appropriate neuronal assemblies^48^. We can speculate that the changes in the activity patterns of vACC neurons described here could facilitate the formation of aberrant neuronal assemblies and, as mentioned above, alter the ability of the ACC to properly encode a pain memory. Certainly, state of the art techniques to monitor the activity of neuronal assemblies in vivo will provide the opportunity to test soon this type of questions.

In conclusion, the experiments presented here demonstrate that neuropathic pain induces an increase in the activity of neurons in the deep layers of the vACC associated with a change in the coding pattern towards burst firing. This has important implications for the processing of nociceptive information in the ACC. This could provide a new cellular target for new treatment strategies for chronic pain.

## Materials and methods

### Animals

Male C57/BL6 mice (Central Animal Facility of the University of Bern or Janvier Labs, France) were housed, grouped, acclimatized to laboratory conditions (12-h light/dark cycles) and had unrestricted access to food and water. For identification, small holes were punched into the mouse ears. All experiments were conducted and approved according to federal guidelines and regulations for animal experimentation of the State Veterinary Department of the Canton of Bern, Switzerland. All methods are reported in accordance with the ARRIVE guidelines.

### Chronic constriction injury and Von Frey test

Sciatic nerve lesions and Von Frey tests were carried on as previously described^23,26,27^. Surgery was performed under a sterile hood. Mice (~ 8 weeks old) were anesthetized with isoflurane (3–4% induction, 1–2% maintenance) vaporized with 100% oxygen, eye ointment (Pharma Medica AG, Rogwil, Switzerland) was applied and fur was shaved from the left thigh. A small incision was performed, the sciatic nerve was exposed and three loose ligatures 1 mm apart were made on the nerve suing a sterile sofsilk thread 5–0 (Coviden, Dublin, Irleand). The skin was then sutured with an absorbable thread (4–0 coated VICRYL rapid suture, Ethicon). Sham animals were subjected to a similar procedure in which the sciatic nerve was exposed but not ligated. Animals did not receive postoperative analgesics, were allowed to recover from the surgery and controlled on a regular basis.

Mechanical sensitization was evaluated with the Von Frey test one day before and 6 days after sciatic nerve surgery. Mice were placed on an elevated grid inside a Plexiglas cylinder and allowed to habituate for ~ 30 min. Mechanical threshold was then tested using an Electronic von Frey aesthesiometer (IITC Life Science, CA, USA) by slowly applying pressure to the midplantar surface of the hind-paw with the Von Frey filament until a paw withdrawal was evoked. Six pressure measures were taken per each paw and an unpaired t-test was used as exclusion/inclusion criterion.

### c-Fos immunohistochemistry

Ten days after sciatic nerve surgery, animals were taken from their homecage and immediately anesthetized with a mix of Ketamine and Xylazine (2 × the regular dose, see below). Then were transcardially perfused with Phosphato Buffer Saline 0.1 M (PBS) followed by 4% paraformaldehyde in PBS. Brains were dissected out, kept overnight in fixative and then in PBS at 4 °C. Coronal sections (50 µm thick) throughout the ACC were cut from tissue block submerged in PBS using a vibratome and stored free-floating in PBS containing 0.1% sodium azide at 4 °C until use.

c-Fos immunostaining was performed in free-floating sections. First, sections were permeabilized with Triton (0.1% in PBS; 3 × 10 min), incubated for 1 h at room temperature in a blocking buffer (PBS containing 5% normal goat serum and 2% Bovine Serum Albumina) and then incubated overnight with the primary antibody (SC-52, rabbit polyclonal; Santa Cruz Biotechnology) in blocking buffer at 4ºC. The next day sections were washed 3 times in 0.1% Triton-PBS and incubated with the secondary antibody conjugated to Alexa Fluor 488 (Invitrogen) in blocking buffer for 3 h at room temperature. Sections were then washed in PBS, mounted on glass slides, stained with Neurotrace 435/455 (Invitrogen) and coverslipped with Vectashield (Vector).

Image stacks (200 µm × 200 µm, ~ 30 µm thick, 2 µm step) were obtained with a confocal microscope (TCS SP2, Leica Microsystems) attached to an upright microscope (DMLFS, Leica) using a 40 × objective (HCX APO L W40x UVI, numerical aperture 0.8, Leica; ^58^) . In each animal, images were taken covering the vACC with a 3 × 3 grid (Fig. 2a). Digital images were processed and analysed with custom-made scripts based on the Matlab Image Processing Toolbox. Briefly, images were low-pass filtered (Wiener method), background (estimated by morphological opening) was subtracted and for each stack, the maximal projection image (MPI) was computed. Finally, for each animal a single image was formed for further analysis, composed of the montage of MPI corresponding to the 3 × 3 grid. To detect c-Fos immuno-reactive cells, the deeper end of layer 2/3 was visually identified and used as a superficial limit of a rectangular region of interest (ROI). Then, ROIs were segmented to find circular objects about the size of cell bodies using circular Hough transform (imfindcircles function in Matlab) and objects with mean fluorescence intensities above 12 times the background standard deviation were considered positive. This method was validated by visual inspection of the images confirming the co-localization of c-Fos immunofluorescence with cell bodies identified with Neurotrace staining.

### In vivo electrophysiology

Recordings were performed between 7 and 21 days after sciatic nerve lesion. Mice were anesthetized with a mix of ketamine and Xylazine (0.1 and 0.01 mg/g respectably, i.*p*.), treated with local anaesthetic (lidocaine) in the scalp and pressure points and secured to a stereotaxic frame. Eye ointment (Pharma Medica AG, Rogwil, Switzerland) was applied and temperature was maintained between 36 °C and 37 °C with a heating pad. Additional Ketamine-Xylazine was administered as required to maintain anaesthesia. A small craniotomy was drilled over the right ACC (0.5–1.2 mm rostral and 0.2–0.8 mm lateral from Bregma) and remaining bones and dura carefully removed. Juxtacellular recordings of ACC neurons (1.2–2 mm below the brain surface) were obtained with glass microelectrodes with a tip impedance of 12–25 MΩ filled with artificial cerebral spinal fluid (in mM; NaCl: 125, NaHCO3: 25, KCl: 2.5, NaH2PO4: 1.25, MgCl2: 145, glucose: 25). Electrode signals were sent to a Dagan BVC-700 amplifier and digitized at 20 kHz with an ITC-16 board (Instrutech) using Igor software (Wavemetrics). An iontophoretic deposit of Pontamine Sky Blue (-100 nA, 20 min) was made at the end of the electrophysiological sessions to use as a reference landmark to reconstruct locations of recorded cells. In some occasions, biocytin (1%) was included in the microelectrodes recording solution to label the cells. For that, positive current pulses (250 ms at 2 Hz) driving spike discharges (3–15 nA) were delivered for ~ 15 min. At the end of the experiment, mice received a high dose of Ketamine-Xylazine and were transcardially perfused with PBS followed by 4% PFA as described above. Labelled neurons were developed with the avidin–biotin-peroxidase method ^26,27^.

To establish juxtacellular recordings, electrodes were slowly descended into the ventral ACC while delivering positive current pulses (500 ms; 200 pA). Upon observing an increase in resistance, the electrode was left in place for at least two minutes, monitoring for neuronal firing. If firing occurred, recording sessions only began when action potentials showed stable signal-to-noise ratio for several minutes. If no action potentials were observed, the electrodes continued to be slowly advanced. Recording sessions consisted of 10 min of spontaneous activity followed by a 45 trials of hind paw electrical stimulation (HPS) at 0.125 Hz. HPS was achieved by applying a brief current (50 ms; 90 V) onto conductive adhesive strips (approximately 1 cm wide by 2 cm long) placed on the hindpaw pad.

Electrophysiological signals were analyzed using custom-made algorithms in MATLAB software environment. Spikes were detected setting a minimum amplitude threshold determined for each cell after visual inspection of the signal. Spike width was measure from an average spike as the time between the start of the rising phase of the spike and the peak of the negative deflection. Firing rate and bursting activity were calculated. To isolate bursting episodes we used a fixed maximum limit threshold method. Under conditions of low firing rate with sparse o little bursting, this strategy performs better than surprise-based methods (i.e. Poisson surprise), which tend to overestimate bursting activity^43^. Thus, a burst was defined as consecutive spikes with inter-spike intervals (ISI) below 30 ms followed by a pause period longer than 100 ms. This ISI limit was calculated from the average ISI histogram as the value corresponding to the half decay of the high frequency firing peak (Fig. 3e). The total number of bursting events, the percentage of total spikes within burst and the intra-burst frequency were calculated within the 10 min of spontaneous activity.

In order to detect neurons responding to electrical HPS, we first calculated a firing rate peri-stimulus histogram with 40 ms bins. We then computed the mean (µ_baseline_) and standard deviation (σ_baseline_) firing rate for 500 ms baseline period before the stimulus onset and a Z score for one second before and after the stimulus, as follows:

$${Z}_{score (bi)}=\frac{{Fr}_{(bi)}-{\mu }_{baseline}}{{\sigma }_{baseline}},$$

where *bi* denotes the bin and FR the firing rate. Bins during the response period (500 ms after stimulus) showing a Z score higher than 2 were considered significantly modulated by HPS. Next, we estimated the response reliability from the number of trials with spikes falling in HPS modulated bins. We considered a neuron to be modulated by HPS if response reliability was higher than 10%. This criterion was chosen because allowed us to exclude cells showing isolated peaks of a high Z score due to a very low firing rate during baseline, while showing a low response reliability. All response properties were measured in HPS-modulated bins: response magnitude was the maximum Z score value; response latency was the time of the first spike after stimulus onset; HPS responses in burst were detected using the same criteria as for spontaneous activity.

### Statistical analysis

Statistical analysis was performed with GraphPad Prism 8 (GraphPad Software, La Jolla, CA, USA) using a critical probability of *P* < 0.05. For Von Frey testing and C-FOS immunostaining experiments, *n* corresponds to the number of animals, while for electrophysiology data *n* corresponds to individual cells collected in 13 mice per group. Data distribution was assessed for normality using D’Agostino & Pearson test. According to this, values are given as means ± SE when data followed a Gaussian distribution or median (1^st^ – 3^rd^ quartile) when did not. Statistical significance between groups for individual variables was tested using t-test or the nonparametric Mann Whitney test, correspondingly. Two-way ANOVA followed by a Bonferroni post test was performed to compare for the mechanical threshold before and after sciatic nerve surgery. The effect size (ES) for statistically significant comparisons was calculated as follows: Mann Whitney test, ES = (n1*n2-U) / (n1*n2); Two-way ANOVA, ES = partial eta squared. In plots showing individual values, horizontal lines correspond to group mean or median values, depending whether data followed a Gaussian distribution or not.
